# Consideration of spatial companion biomarkers for targeted therapeutics in cancer: depatuxizumab mafodotin in glioblastoma

**DOI:** 10.1172/jci.insight.198475

**Published:** 2026-02-03

**Authors:** Rimas V. Lukas, Ruochen Du, Harrshavasan Congivaram, Kathleen McCortney, Karan Dixit, Craig Horbinski, Margaret Schwartz, Raymond Lezon, Lauren Singer, Ditte Primdahl, Jigisha Thakkar, Amy B. Heimberger, Roger Stupp, Priya Kumthekar

**Affiliations:** 1 Department of Neurology, and; 2 Malnati Brain Tumor Institute, Northwestern University, Chicago, Illinois, USA.; 3 Department of Pathology, and; 4 Department of Neurological Surgery, University of Chicago, Chicago, Illinois, USA.; 5 Department of Neurology, and; 6 Department of Neurology, Loyola University, Chicago, Illinois, USA.

**Keywords:** Neuroscience, Oncology, Brain cancer

## Abstract

An expanded access program of depatuxizumab mafodotin for patients with progressive glioblastoma explores the relationship between tumor EGFR expression and outcomes.

**To the Editor:** Glioblastoma is a malignant primary central nervous system tumor with frequent epidermal growth factor receptor (EGFR) pathway aberrancies, including amplification and/or mutation of the *EGFR* gene and overexpression of the cell surface receptor, thereby serving as an enticing target in those tumors where these aberrancies are detected. In newly diagnosed glioblastoma, EGFR amplification is present in approximately 50% of glioblastomas, with concurrent expression of tumor-specific mutant *EGFRvIII* in approximately half of amplified cases (1), with a less clear understanding of its aberrations in progressive tumors. Unfortunately, targeting EGFR has been unsuccessful thus far (2). The EGFR-targeting antibody-drug conjugate, depatuxizumab mafodotin (ABT-414), is composed of a humanized chimeric recombinant IgG1 κ EGFR-specific antibody attached to the antimitotic cytotoxic monomethylauristatin F via a maleimidocaproyl linker. ABT-414 can toggle binding to activated, amplified wild-type EGFR or EGFRvIII, rendering it tumor preferential but not specific. A phase III trial for EGFR-amplified newly diagnosed glioblastoma added ABT-414 to radiotherapy and temozolomide. Progression-free survival (PFS) was longer, but with no increase in survival (3).

Spatial localization of EGFR relative to the site of vascular entry into the tumor has not been previously considered. There is marked tumor heterogeneity in vascular size and distribution within glioblastomas. Intravenously administered antibodies, as a function of molecular weight and tissue flow kinetics, have limited diffusion. Furthermore, longitudinal loss of EGFRvIII expression contributes to lack of therapeutic effects for peptide vaccines and chimeric antigen receptor T cell strategies.

Using a single institutional expanded access program (EAP) of ABT-414 approved by the IRB at Northwestern University (NU17CU02, NCT03123952; see the “Summarized inclusion criteria” and “Expanded Access Protocol” in the supplemental material; supplemental material available online with this article; https://doi.org/10.1172/jci.insight.198475DS1), analysis was conducted on spatial heterogeneity of EGFR relative to vasculature in progressive glioblastoma. ABT-414 (1.25 mg/kg i.v.) was administered every 2 weeks as long as tolerated and efficacious. The EAP allowed for ABT-414 in combination with additional FDA-approved glioblastoma treatments, providing real-world experience.

Twenty-nine heavily pretreated patients (Supplemental Table 1) were enrolled in the EAP with a median age of 55 years (36–68 years), majority male (*n* = 20, 69%) and White (*n* = 26, 90%). Patients were treated with ABT-414 monotherapy (*n* = 13), or in combination with temozolomide (*n* = 7), CCNU (*n* = 3), bevacizumab (*n* = 3), temozolomide plus bevacizumab (*n* = 2), and temozolomide plus tumor-treating fields (*n* = 1). Participants received between 1 (*n* = 10 patients) and 44 doses, with 24% receiving 20 or more doses of ABT-414. Most (*n* = 28, 97%) developed treatment-related adverse events (AEs). Grade 3/4 AEs were common (*n* = 22, 76%), and one grade 5 AE (3%) was observed. The most common treatment-related AEs were thrombocytopenia (66%) and blurred vision (35%), aligning with what has been previously reported. No new unexpected AEs were identified, indicating that the experience with ABT-414 for patients with recurrent glioblastoma within an EAP demonstrated toxicity similar to what has been observed in previously published trials.

Of 29 patients treated, 23 were evaluable for radiographic responses. No responses were observed. Approximately half (*n* = 11, 48%) demonstrated stable disease, while the other half (*n* = 12, 52%) had progressive disease. Median survival was 6.7 months (95% CI, 4.07–10.2 months) (Figure 1A) and median PFS was 2.4 months (95% CI, 1.9–5.6 months) (Supplemental Figure 1).

Ad hoc retrospective analysis of spatial EGFR expression was performed on a subset of patients for whom the original resection blocks could be obtained (Supplemental Dataset 1). Cell segmentation, background autofluorescence subtraction, and thresholding were conducted using an established bioinformatic pipeline analysis. EGFR expression was heterogeneous, including tumor regions devoid of expression (Figure 1B). Total expression at the time of the initial diagnosis was in the range of 11%–91% (*n* = 6) and did not associate with PFS. GFAP^+^ tumor cells abutted vasculature but also extended far into the parenchyma (Figure 1, C and D). In some instances, GFAP^+^ tumor cells surrounding the vasculature were EGFR^+^ (Figure 1E), whereas other perivascular regions lacked EGFR expression (Figure 1F), indicating tumor cells would persist even in the presence of an EGFR-targeting moiety administered intravenously. Presence of EGFR^+^ tumor cells adjacent to the vasculature was not associated with PFS (Supplemental Figure 2).

Antibody penetration distance is limited by clearance and antigen turnover rate. For a 70 kg patient with blood volume of 5.5 L, the circulating concentration of ABT-414 would be approximately 110 nM (roughly 1 × 10^–7^ mol/L), corresponding to an estimated penetration distance of approximately 15–40 μm, depending on EGFR turnover (4, 5). Spatial quantification analysis using a diffusion penetration distance of 40 μm indicates only 24%–68% of total cells would be in range of a vessel to have been exposed to ABT-414 (*n* = 6). This level of cytotoxicity is likely well below the threshold needed to impact survival for glioblastoma. These findings illustrate that dichotomized readouts for biomarkers are insufficient in heterogeneous diseases and that companion biomarkers should consider spatial target expression and antibody distribution. Selection of highly vascular tumors and/or delivery strategies, including blood-brain barrier opening ultrasound and convection-enhanced delivery, could address therapeutic tumor distribution. Additionally, antibody-drug conjugates developed with cleavable linkers (unlike ABT-414) could mediate bystander via release of payload, leading to improved penetration distance and potentially improved potency. However, uniform target expression remains a continued challenge for many solid cancers. Whether the resected tumor is reflective of the residual/progressive disease state during treatment is a significant knowledge gap needing to be addressed, especially for targeted therapy strategies.

## Funding support

This work is the result of NIH funding, in whole or in part, and is subject to the NIH Public Access Policy. Through acceptance of this federal funding, the NIH has been given a right to make the work publicly available in PubMed Central.

## Supplementary Material

Supplemental data

Supplemental data set 1

undefined

## Figures and Tables

**Figure 1:**
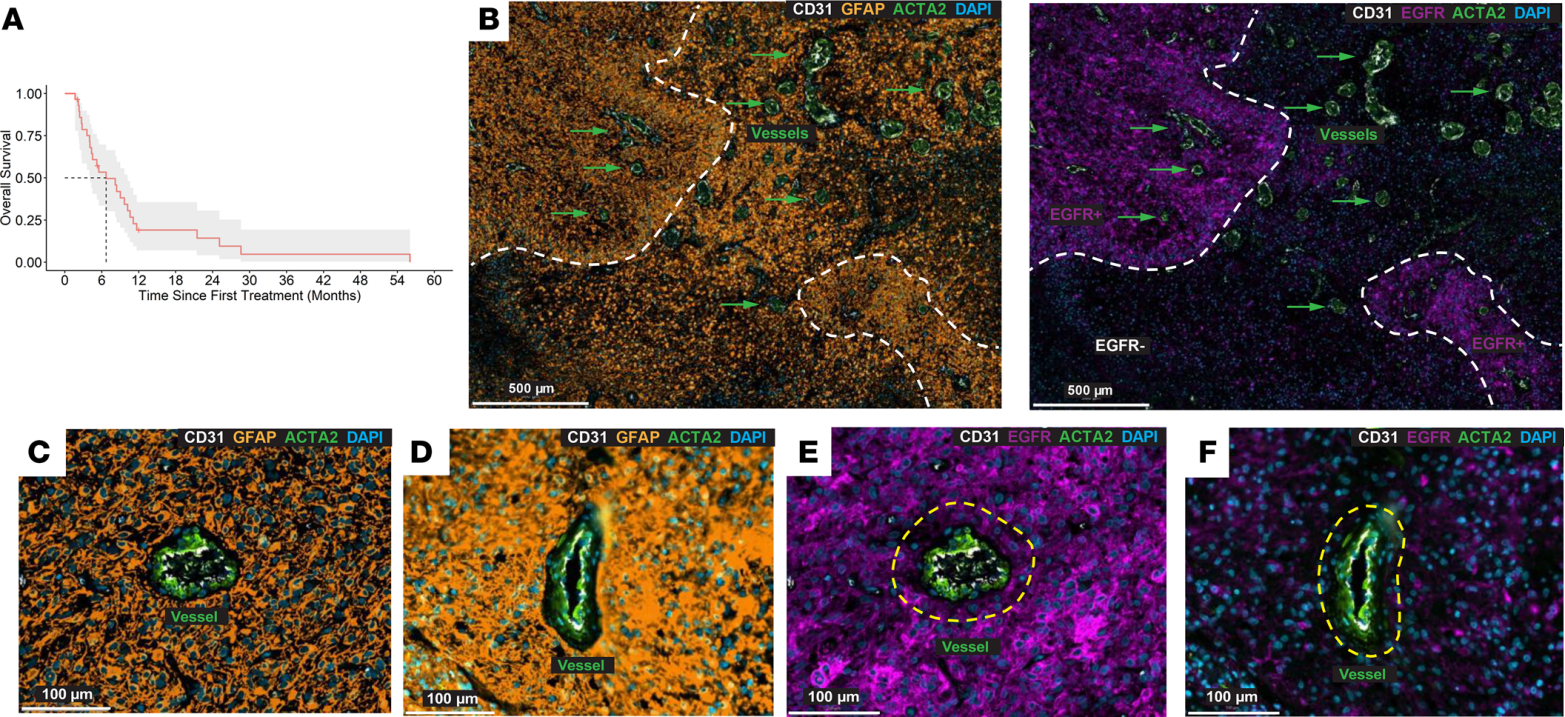
(**A**) Kaplan-Meier survival curve, showing a median overall survival of 6.7 months (95% CI, 4.1–10.2 months). (**B**) Representative spatial sequential immunofluorescence (SeqIF) images demonstrating heterogeneity of EGFR expression within GFAP^+^ glioblastoma. The white lines demarcate EGFR protein expression within the solid tumor region. Green arrows designate vessels. (**C** and **D**) Representative spatial SeqIF images of GFAP^+^ tumor cell expression (pseudocolored orange) surrounding ACTA2^+^ (pseudocolored green) and CD31^+^ (pseudocolored white) vessels from 2 different tumors. (**E** and **F**) Matched spatial SeqIF images of EGFR^+^ tumor cell expression (pseudocolored purple) surrounding ACTA2^+^ and CD31^+^ tumor vessels. (**E**) Demonstrates homogeneous EGFR expression in proximity to the vessel, while (**F**) shows minimal EGFR expression surrounding the tumor vasculature. Yellow dashed lines designate the perivascular diffusion penetration distance quantified at 40 μm. Scale bars: 500 μm (**B**) and 100 μm (**C**–**F**).

## Version 1 02/03/2026

In-Press Preview

## Version 2 03/23/2026

Electronic publication
