# Supplementary material for: Consideration of spatial companion biomarkers for targeted therapeutics in cancer: depatuxizumab mafodotin in glioblastoma
Source: JCI Insight. 2026 Feb 3;11(6):e198475. doi: 10.1172/jci.insight.198475 (PMC13043081; doi:10.1172/jci.insight.198475)

SUPPLEMENTAL MATERIAL: Consideration of spatial companion biomarkers for targeted therapeutics in cancer: depatuxizumab mafodotin in glioblastoma

Sex as a biological variable.

Both male (n=20) and female (n=9) patients were included in this study. The distribution observed in our study is in line with what would be expected. There is a slightly higher incidence of glioblastoma in men compared to women.

Statistics overview.

Kaplan-Meier method was used to develop curves for overall survival (OS) and progression free survival (PFS). A scatter plot was used to analyze the relationship between perivascular EGFR+ tumor cells and PFS.

Study approval.

This study was approved by the Northwestern University Institutional Review Board (IRB).

Data Availability.

This human subject data is not publicly available. This data may be made available in anonymized fashion upon request and within the context of an IRB approved protocol. Values for all data points in graphs are reported in the Supporting Data Values file.

Summarized inclusion criteria.

Inclusion criteria: ≥18 years old, *EGFR* amplification or *EGFRvIII* mutation, stable/decreasing steroids, absolute neutrophils ≥1,500/mm^3, platelets ≥75,000/mm^3, (≥100,000/mm^3 with temozolomide), and hemoglobin ≥9.0 gm/dL. Patients who were pregnant, had severe hepatic impairment, or had an infection were excluded. Patients underwent pre-treatment ophthalmologic assessment, and then, as indicated.

Expanded Access Protocol. Expanded Access use of ABT-414 in Recurrent Glioblastoma Multiforme (GBM)

Protocol

NU 17CU02

IND Number: 136218

Amendment 4 dated 08Jan2019

Principal investigator

Priya Kumthekar, MD

Northwestern University Feinberg School of Medicine
Northwestern Medicine

675 N Saint Clair Street
Suite 21-100

Chicago, IL 60611

Tel: 312-695-0990

Fax: 312-695-1435

Pkumthe2@nm.org

Expanded Access use of ABT-414 in Recurrent Glioblastoma Multiforme (GBM)

PI: Priya Kumthekar, MD

Sponsor: Priya Kumthekar, MD (Northwestern Medicine)

Manufacturer: AbbVie, One North Waukegan Rd**,** North Chicago, IL 60064

1. INTRODUCTION

1.1 Indication:

Adult patients with recurrent GBM who have EGFR amplification or have an EGFRvIII mutation.

1.2 Background:

1.2.1 Glioblastoma Multiforme (GBM)

GBM is the most common and most aggressive type of primary brain tumor in adults, affecting 8,000 to 10,000 people per year in North America alone [1]. The median survival time from diagnosis is 3 months without any treatment. With treatment, patients can expect a survival of 1 to 2 years. The survival depends greatly on many patient and tumor specific factors, including tumor stage, age, resectability, and genetic characteristics as well as performance status. Treatments may include surgical resection of the tumor, chemotherapy, radiation therapy, and immunotherapy. Despite advances in treatments, the prognosis of patients with GBM remains poor.

The current standard-of-care therapy for newly diagnosed glioblastoma following surgical debulking is radiation therapy (RT) in combination with temozolomide (TMZ), followed by 6 months of further temozolomide monotherapy [2]. The standard of care is based on the results from a definitive Phase 3 trial conducted by the European Organization for Research and

Treatment of Cancer (EORTC) and NCI of Canada (NCIC) that demonstrated a survival benefit for patients treated with temozolomide in addition to surgery and radiation [3]. In this study, patients in the radiation therapy plus concomitant and adjuvant temozolomide arm had a longer median survival time (14.6 versus 12.1 months) and a greater 2-year survival rate (27% versus 10%) than patients in the radiation therapy alone arm.

Patients with GBM eventually have recurrent disease with a dismal prognosis. TMZ remains the most common treatment for the recurrent GBM. TMZ was compared with procarbazine in patients with first relapsed GBM in a randomized Phase 2 trial. The 6-month PFS rate was 21% versus 8%, median PFS was 12.4 weeks versus 8.32 weeks, and the 6-month survival rate was 60% versus 44% in TMZ versus procarbazine arms, respectively [4].

GBM tumors have unique molecular characteristics that can influence outcome and inform treatment decisions. O6-methylguanine-methyltransferase enzyme (MGMT) silencing has been shown to significantly affect the response to temozolomide (TMZ) therapy [5]. The maximum benefit of temozolomide treatment is limited to the proportion of patients (approximately 45% in the EORTC/NCIC Phase 3 trial discussed above) whose tumors have methylation-induced silencing of the MGMT gene [6]. Phosphatase and tensin homolog (PTEN) and EGFR expression have also correlated with outcome measures. GBM tumors, particularly those with EGFR amplification, have a high rate of EGFRde2-7 mutation expression (up to 40% in the overall population and up to 60% of more in the EGFR amplification population). The presence of this mutation confers a worse prognosis [7].

1.2.2 ABT-414

ABT-414 is an antibody-drug conjugate (ADC) composed of the anti-EGFR antibody ABT-806 conjugated via a non-cleavable maleimidocaproyl (mc) linker to a potent anti-mitotic cytotoxin, monomethylauristatin F (MMAF). ABT-806 is a humanized chimeric recombinant IgG1κ antibody specific to a unique epitope of human EGFR accessible in tumors expressing EGFRde2-7/EGFRvIII deletion mutants and in tumors with activated wild-type amplified EGFR. The antibody component of ABT-414 delivers MMAF specifically to antigen-positive cells, where it disrupts the microtubule network inducing cell cycle arrest and cell death. ABT-414 binds a unique epitope of EGFR, which is largely inaccessible when EGFR is expressed at physiological levels. Rather, the targeted epitope is accessible in tumors that express EGFR with deletion of exons 2 through 7 (EGFRde2-7, also referred to as EGFR variant III [EGFRvIII]) and in tumors with activated wild-type amplified EGFR. Thus, ABT-414 has tumor-specific binding properties. ABT-414 is highly effective in both mutant and wild-type EGFR-positive human tumor xenografts in animal models, supporting its development in patients with EGFR-positive malignancies.

1.2.3 Targeting the Epidermal Growth Factor Receptor (EGFR)

The epidermal growth factor receptor (EGFR) plays a causal role in the development and maintenance of many human carcinomas. Overexpression, activation, and mutation of the EGFR in epithelial tumors are associated with aggressive cancer phenotypes and have been implicated in tumor progression via anti-apoptosis, cellular proliferation, angiogenesis, and metastasis.

EGFR became a validated oncology target upon the first clinical trials with EGFR-directed monoclonal antibodies, e.g., cetuximab and panitumumab, as well as EGFR-directed tyrosine kinase inhibitors, e.g., erlotinib and gefitinib. These EGFR-directed therapies have gained widespread use for a broad variety of different cancer types, including lung cancer, head and neck cancer, colon cancer and pancreatic cancer. In many cases, these EGFR-directed therapies have shown improvements in both progression-free survival and overall survival while preserving quality of life.

One of the unique molecular characteristics of GBM tumors is the aberrant signaling, expression, mutation, and gene amplification of the epidermal growth factor receptor, or EGFR. The EGFR has been hypothesized to play a critical role in the development of GBM and thus has an extremely high rate of overexpression (nearing 60% or more), amplification (approximating 50%), and mutations (at least 25% have been reported to harbor the constitutively active EGFR variant III (EGFRvIII) mutation). This makes the EGFR an attractive target for drug therapy. Unfortunately, EGFR signaling inhibitors have not been successful to date, likely due to the multiple signaling bypass pathways as well as the underlying and reactive mutational resistance mechanisms, and limited brain penetration of tyrosine kinase inhibitors at routine dosing used in other solid tumors. Other methods to exploit the EGFR, such as using the receptor as a target for selective chemotherapy or immunotherapy delivery, remain attractive.

ABT-414 is an antibody drug conjugate (ADC) designed for the treatment of tumors with elevated levels of EGFR protein. Antibody drug conjugates are a rapidly growing class of cancer drugs that combine the targeting properties of monoclonal antibodies (mAbs) with the anti-tumor effects of potent cytotoxic drugs. A recent example of a clinically relevant, advanced ADC is brentuximab vedotin (Adcetris®), an anti-CD30 ADC which received FDA approval in 2011 for Hodgkin's lymphoma and anaplastic large cell lymphoma due to a high percentage of tumor responses in these largely chemotherapy refractory populations. The distinct clinical advantage to ADCs is their ability to deliver cytotoxic payloads directly to a tumor, bypassing many downstream resistance mechanisms related to intracellular signaling.

ABT-414 is a newer generation ADC, consisting of: 1) a veneered "humanized" recombinant IgG1κ antibody that has binding properties specific to a unique epitope of human EGFR with 2) non-cleavable maleimido-caproyl linkers each attached to 3) a potent antimicrotubule agent,

monomethylauristatin F (MMAF). ABT-414 binds to an epitope that is available predominantly on tumor cells with the EGFRde2-7 (EGFRvIII) deletion mutant or on tumor cells with activated wild-type EGF receptors. The epitope is largely inaccessible when EGFR is expressed at normal physiological levels; thus, ABT-414 has limited binding to non-activated, wild-type EGFR expressed on normal tissues. These properties, therefore, favor limited effects of the toxin on normal tissues while maintaining a high degree of activity on EGFR-overexpressing tumor cells.

1.3 Risks and Benefits:

ABT-414 is an antibody-drug conjugate (ADC) that has demonstrated both preclinical and preliminary clinical efficacy in GBM.

In preclinical studies, ABT-414 has demonstrated efficacy in a range of tumor types, including human glioblastoma cell lines that have a high EGFR expression, and human tumor xenograft models. ABT-414 also demonstrates a favorable EGFR binding ratio of tumor to normal tissue.

ABT-414 has been studied in 5 clinical trials to date. Study M13-379 is a first-in-human study in patients with advanced solid tumors. Study M12-356 is a Phase 1 study in patients with GBM (recurrent and newly diagnosed). Study M14-483 is a Phase 2 study in patients with recurrent EGFR-amplified GBM. Study M13-714 is a Phase 1/2 study in Japanese patients with high grade glioma. And Study M13-813 is a Phase 2/3 study in patients with newly diagnosed EGFR-amplified GBM. Preliminary efficacy data are encouraging, particularly for those with tumors that are either EGFR gene amplified or harbor an EGFRvIII deletion. As of 20 April 2016, preliminary safety data were available for 377 subjects who received at least 1 dose of study drug in these 5 clinical studies conducted to date. The most frequent treatment emergent adverse event was related to the development of corneal epithelial microcysts, a toxicity that has been described for other antibody-drug conjugates, including those that contain MMAF. The most common non-ocular adverse events in these studies include myelosuppression, fatigue, and neurological events such as seizure. The most common Grade 3 or 4 toxicities considered to have a relationship to the study drug were eye disorders, as well as, fatigue and increased liver function variables (ALT and AST).

Together, these data reflect an acceptable rationale and risk for treating patients with cancer that has a moderate to high level of EGFR expression with ABT-414 in the context of this expanded access use.

Adverse Events that are known, and those not previously described, may occur with ABT-414 as detailed in the consent to treat for this study. In addition, patients may experience inconvenience or discomfort related to the visits or procedures.

1.4 Patient Population:

Given the high frequency of EGFR amplification in GBM, as well as clinical and preclinical data obtained to date, this expanded access protocol will allow access to therapy that might show clinical benefit to patients diagnosed with a life-threatening disease (GBM) who do not qualify for an ABT-414 clinical trial. The efficacy to date has been limited to those with EGFR amplification; thus it is strongly recommended that only those with amplification of EGFR be treated with ABT-414 under this protocol. Because (a) EGFRvIII mutation is seen almost exclusively in the context of EGFR amplification and (b) the antibody portion of ABT-414 has high affinity for the EGFRvIII mutation, it also is appropriate for patients who have EGFRvIII-mutated tumors to receive ABT-414 if EGFR amplification testing results are not available.

1.5 Clinical Data of ABT-414 in GBM:

ABT-414 has been studied in 5 clinical trials to date. Study M13-379 is a first-in-human study in patients with advanced solid tumors. Study M12-356 is a Phase 1 study in patients with GBM (recurrent and newly diagnosed). Study M14-483 is a Phase 2 study in patients with recurrent EGFR-amplified GBM. Study M13-714 is a Phase 1/2 study in Japanese patients with high grade glioma. And Study M13-813 is a Phase 2/3 study in patients with newly diagnosed EGFR-amplified GBM. As of 20 April 2016, preliminary safety data were available for 377 subjects who received at least 1 dose of study drug in these 5 clinical studies conducted to date.

1.5.1 Clinical Pharmacokinetics

Early pharmacokinetic results for 68 subjects with GBM in Study M12-356 showed that the harmonic mean terminal half-lives of ABT-414, total ABT-806, and Cys-mcMMAF are approximately 7, 9, and 4 days, respectively. Cys-mcMMAF was not a direct, time- or metabolism-dependent inhibitor of CYP1A2, CYP2B6, CYP2C8, CYP2C9, CYP2C19, CYP2D6, or CYP3A4/5, nor an inducer of CYP1A2, 2B6, or 3A/4/5 enzyme activity or gene expression and thus, is unlikely to have drug-drug interaction in combination studies. Overall, the systemic exposures (AUC and Cmax) of ABT-414, total ABT-806, and Cys-mcMMAF achieved after administration of ABT-414 via IV infusion appeared to be approximately dose proportional. Preliminary data suggest that ABT-414 (0.5 to 1.5 mg/kg dose range) has no influence on the pharmacokinetics of TMZ. The effect of TMZ (150 to 200 mg/m2) on ABT-414 pharmacokinetics was evaluated by comparing Cmax and AUC between Arm B and Arm C. At 1.25 mg/kg ABT-414, ABT-414 and Cys-mcMMAF pharmacokinetic parameters are comparable with and without TMZ, indicating that TMZ has no effect on ABT-414 pharmacokinetics.

1.5.2 Clinical Safety

Eye toxicities are the most common treatment-related adverse events observed, and they define the dose limiting toxicity (DLT) for ABT-414. These events have been manifestations of the development of corneal epithelial microcysts, referred to as microcystic keratopathy. Adverse events associated with microcystic keratopathy include a variety of eye-related signs (including keratitis, corneal cysts, corneal deposits, ocular hyperemia) and symptoms (most commonly vision blurred, photophobia, dry eye, eye pain, foreign body sensation in eyes, and lacrimation increased).

Standard prophylaxis with ocular steroids appeared to decrease the severity of symptoms related to microcystic keratopathy and allow subjects to tolerate higher doses of ABT-414. Although ocular steroid prophylaxis may improve tolerability of ABT-414, it does not appear to decrease the overall incidence of microcystic keratopathy. To date, all cases of microcystic keratopathy have been reversible without long-term sequelae, usually 4 to 6 weeks after ABT-414 discontinuation. Dexamethasone 0.1% solution, prednisolone acetate 1% suspension, or an equivalent steroid ophthalmologic solution, is recommended around the time of each ABT-414 infusion.

Overall, the majority of nonocular adverse events seen in subjects receiving ABT-414 are events that are anticipated to occur commonly due to either the underlying illness or concomitant chemotherapy or chemoradiotherapy including myelosuppression, fatigue, and neurological events such as seizure. Other than eye toxicity related to corneal microcystic keratopathy, there have been no consistent, clinically significant safety findings suggesting a causal relationship with ABT-414, either as monotherapy or in combination with other chemotherapy.

1.5.3 Clinical Efficacy

There have been encouraging reports of clinical benefit in past and current ABT-414 clinical trials with both ABT-414 monotherapy and in combination with TMZ. In addition, an independent data monitoring committee recently conducted a planned futility analysis for Study M14-483 (Phase 2 EGFR-amplified recurrent GBM patients) and recommended the study continue without any changes. All subjects with confirmed responses to therapy had tumors with evidence of EGFR amplification, and none of the subjects with tumors negative for EGFR amplification has experienced a confirmed objective response.

A detailed discussion of the preclinical toxicology, metabolism, and pharmacology can be found in the Investigator Brochure.

1.6 Rationale:

The distinct clinical advantage of ABT-414 is its ability to deliver a toxic payload to a tumor cell, bypassing downstream resistance mechanisms related to intracellular signaling that are encountered with inhibitors of EGFR functions.

Importantly, ABT-414 binds to an epitope that is available predominantly on tumor cells with the EGFRde2-7 deletion mutant or on tumor cells with activated wild-type EGF receptors, and in the context of EGFR gene amplification. The epitope is largely inaccessible when EGFR is expressed at normal physiological levels as demonstrated in previous trials of the unconjugated antibody (ABT-806), where acneiform rashes were infrequent and mild at doses up to 4 to 6 times higher than those of marketed EGFR antibodies. Thus, ABT-414 has limited binding to nonactivated, wild-type EGFR expressed on normal tissues. These properties, therefore, should limit the effects of the toxin on normal tissues while maintaining a high degree of activity in EGFR - overexpressing tumor cells.

2. OBJECTIVE

2.1 Primary Objective:

The primary object is to provide ABT-414 under the Expanded Access Program for up to 40 patients with recurrent GBM who has EGFR amplification or has an EGFRvIII mutation. The patient may continue to receive ABT-414 until she/he is unable to tolerate the drug or the patient has progressive/recurrent disease.

3. PATIENT ELIGIBILITY

Up to 40 patients may be enrolled if they meet all of the Inclusion Criteria and do not meet any of the Exclusion Criteria.

3.1 Inclusion criteria:

1. The patient has a histologic diagnosis of GBM with either EGFR amplification or EGFRvIII mutation.
2. Adult ≥ 18 years of age.
3. The patient is being treated in the recurrent setting.
4. The patient would not otherwise be eligible for an ABT-414 clinical trial.
5. The patient has adequate hematologic and renal function as follows:

- Absolute neutrophil count (ANC) ≥ 1,500/mm^3;

- Platelets ≥ 100,000/mm^3 when administering ABT-414 in combination with temozolomide;
- Platelets ≥ 75,000/mm^3 when administering ABT-414 monotherapy;
- Hemoglobin ≥ 9.0 g/dL (transfusion to achieve Hemoglobin ≥ 9.0 g/dL is acceptable);
- Serum creatinine ≤ 1.5 times the upper limit of the normal range.

6. Patient is receiving stable or decreasing doses of steroids for 7 days prior to enrollment
7. If the patient is female and of childbearing potential, or if the patient is male and non-sterile, the patient agrees to practice with partner(s) at least one of the methods of birth control listed in Appendix A beginning with initial ABT-414 administration and continuing until 65 days (for women) or 125 days (for men) after last dose of ABT-414.

3.2 EGFR Amplification Testing

Tumor samples will be tested for EGFR amplification and expression at Northwestern pathology laboratories. The tumor sample must test positive for EGFR amplification in order for the patient to be eligible for enrollment in the expanded access protocol.

3.3 Exclusion criteria:

1. The patient is pregnant or breastfeeding.
2. The patient has severe hepatic impairment per treating investigator's opinion.
3. Patient has active uncontrolled infection

3.4 Registration:

Patients will be registered to the expanded access protocol as outlined below:

1. Potential patient identified by clinic team and coordinator notified.
2. Patient consented.
3. Review and confirmation of eligibility, including scheduling of any baseline exams or tests needed for screening; treating physician will sign off on final eligibility checklist once all screening procedures are complete.
4. Coordinator registers the patient in NOTIS and sends physician request form to Abbvie.
5. Once registered, coordinator will notify the team.
6. Treatment may be scheduled once registration is complete and approval is received from the Abbvie medial monitor.

4. TREATMENT PLAN

4.1 Pre-Treatment Evaluation:

The patient will be evaluated by his/her physician prior to starting treatment with ABT-414 under direction of this expanded access protocol.

The physician will evaluate the patient's laboratory data and clinical condition (based on physical examination) immediately before the first dose of ABT-414. This initial evaluation will serve as screening to determine if the patient is appropriate to initiate treatment with ABT-414.

Screening evaluations will be completed within 4 weeks prior to treatment (unless otherwise noted below) and should include:

- Physical Examination
- Neurologic Examination
- Medical and Oncologic History
- Vital signs (weight, blood pressure, heart rate and temperature)
- Urine or Serum pregnancy test within 14 days for women who are able to have children)
- Routine laboratory tests including tests of blood and urine (within 14 days)
- Ophthalmology Exam
- EGFR expression

A baseline ophthalmology exam will be performed for all patients prior to initiating treatment. This exam will include the following: external examination, visual acuity, amplitude of accommodation, color vision, cover test, stereopsis, near point of convergence, extraocular motilities, pupils, visual field screening (Humphrey Field Test if available) and interpupillary distance. Other testing may also be performed at the discretion of the examining physician. Additional exams may be performed during the treatment as clinically indicated (e.g., symptoms of dry eyes). In particular, patients developing ophthalmologic toxicities must have an examination by an ophthalmologist, including a thorough slit-lamp exam, to evaluate for the presence of epithelial microcysts.

4.2 Evaluation During Treatment:

The treating physician will regularly evaluate the patient during treatment with ABT-414 to monitor safety, response and/or disease progression. The patient will be seen clinically by the treating physician or designee every four weeks before ABT-414 infusion and evaluations will be performed as outlined below. A complete blood count (CBC) will be performed prior to each infusion and patients must meet platelet and ANC cut-offs as defined in eligibility (ANC ≥ 1500/mm^3 and platelets ≥ 100,000mm^3). Radiological evaluations will be made every 8 weeks (+/- 7 days) using brain MRIs. Additional tests should be added if new symptomatology

warrants additional investigation.

The following procedures will be performed (the window for completion will be +/- 7 days unless otherwise noted):

- Physical Examination including weight – every 4 weeks
- Neurologic Examination – every 4 weeks
- Vital Signs (blood pressure, heart rate, and temperature) – prior to every ABT-414 infusion
- Chemistry (CMP including AST, ALT, and bilirubin) – every 4 weeks
- Hematology (CBC) – up to 3 days prior to every ABT-414 infusion
- Urinalysis – every 4 weeks
- Ophthalmology exam - As clinically indicated
- Karnofsky Performance Status (KPS) – every 4 weeks
- Adverse Event Assessment/Concomitant Medications – at every ABT-414 infusion
- MRI with Contrast – Every 8 weeks
- ABT-414 Administration – Every 2 weeks (+/- 2 days)

4.3 Concomitant Therapy Administration:

Patients may receive additional standard of care therapies in combination with ABT-414 if deemed appropriate by the treating physician and approved by the PI and the Abbvie medical monitor.

For patients receiving concomitant temozolomide therapy, temozolomide will be administered orally once per day for 5 consecutive days (Days 1 – 5) of a 28-day cycle or as per local prescribing information.

The starting dose for the first cycle will be 150 mg/m²/day. The dose of temozolomide can be escalated per treating physician's discretion to 200 mg/m²/day in the second cycle and subsequent cycles.

For patients receiving concomitant bevacizumab, patients will receive bevacizumab infusion immediately prior to ABT-414 infusion, at a dose of 10 mg/kg IV every 2 weeks per local institutional guidelines. The dose will remain stable unless there is a > 10% increase in weight from baseline. The initial dose will be delivered over a period of 90 +/-15 minutes. Infusion protocols and timelines should follow local institutional practices per treating physician's discretion; additional monitoring of urine protein may be required per local institutional practice.

4.4 ABT-414 Dosing and Treatment Administration:

ABT-414 Dose 1.25 mg/kg IV will be administered every 2 weeks by intravenous infusion over 30 to 40 minutes as monotherapy or in combination with another standard of care agent, at the discretion of the physician.

The patient will be closely monitored for treatment-related adverse events, especially allergic reactions, during the infusion. Pre-infusion vital signs should will be taken for each infusion. Direct observation and monitoring post-infusion are required for the first 60 minutes following the initial ABT-414 infusion; post-infusion monitoring is not required for subsequent infusions unless infusion reactions are noted and such monitoring is deemed necessary by the treating physician. Severe allergic reactions (Grade 3 or Grade 4) require the immediate interruption of ABT-414 treatment and discontinuation from the program. Moderate allergic reactions (Grade 1 or Grade 2) will also require the immediate interruption of ABT-414 treatment. Once symptoms have resolved, administration of ABT-414 may continue, at a 60 to 70 minute infusion rate. All subsequent infusions will be administered over 60 to 70 minutes.

Due to the risk of eye toxicity, each administration of ABT-414 Dose 1.25 mg/kg IV should be given with steroid ophthalmic solution. The recommended type, dose, and schedule of eye drops is as follows: dexamethasone 0.1% solution or prednisolone acetate 1.0 % suspension, 2 drops (gtts) in each eye (OU) every 8 (q8) hours to start 48 hours prior to ABT-414 dosing and continue for a total of 7 days (or 21 doses total). The type of ophthalmic solution used may vary depending on the availability of the solution at each location. A modification to the eye drop dosing or schedule based on ongoing clinical experience may be suggested.

Please refer to Section 6.3 for additional toxicity management guidelines. Any dose modifications to ABT-414 for toxicity management will be per treating physician's discretion with approval from the PI. Dose modification levels for ABT-414 are as follows:

Dose Level	*Dose*
Starting dose	*1.25 mg/kg*
1st reduction	*1.0 mg/kg*
2nd reduction	*0.75 mg/kg*
3rd reduction	*0.5 mg/kg*
After 3rd reduction	*Discontinue*

4.5 Off-Treatment Criteria:

The patient will be taken off treatment for the following reasons:

- The patient experiences either clinical or radiographic progressive disease. The patient may continue/resume ABT-414 treatment if subsequent clinical information

suggests that

disease progression has not occurred (e.g., surgical resection histology shows no evidence of tumor).

- The patient develops a severe allergic reactions (Grade 3 or Grade 4).
- The patient develops other toxicities that prohibit further use or intolerable adverse effects.
- The physician believes it is in the best interest of the patient.
- Clinically significant deterioration of the patient's medical status as determined by the physician.
- The patient requires alternative anti-cancer agents that are not compatible with concurrent ABT-414 administration (per treating physician discretion).
- Patient becomes pregnant or begins breastfeeding during the treatment portion of the study.
- The patient decides to withdraw consent for any reason.
- The patient demonstrates noncompliance with the protocol required schedule of treatment visits and procedures.
- Any other medical reason that the physician deems appropriate.
- The ABT-414 expanded access program can also be discontinued by AbbVie

4.6 Off-Treatment Evaluation:

Once the patient is discontinued from treatment, a final visit may be conducted approximately 30 days post-last ABT-414 infusion (+/- 7 days) and the following procedures performed:

- Complete physical examination including weight
- Neurologic Examination
- Vital Signs (blood pressure, heart rate, and temperature)
- Routine blood tests (CBC and chemistry panel)
- Toxicity assessment
- Ophthalmology exam (as clinically indicated)

4.8 Other Concomitant medications:

With the exception of agents listed in Section 4.3, no anti-cancer agents, anti-cancer medicinal/herbal remedies, investigational agents or anti-cancer hormonal therapy may be taken concurrently with treatment. Hormonal contraceptives and hormonal replacement therapy are allowed. Anticonvulsants may be used as clinically indicated. Corticosteroids may be administered at the treating physician's discretion. Prophylactic antiemetics may be administered at the doctor's discretion.

Both corticosteroid therapy and continuous temozolomide therapy induce lymphocytopenia. Subjects receiving either of these drugs or both concomitantly are at an increased risk for opportunistic infections. Therefore, a prophylaxis against Pneumocystis carinii for all subjects receiving temozolomide during radiation therapy such as trimethoprim-sulfamethoxazole (Bactrim forte®, Bactrim DS®), monthly pentamidine inhalations or dapsone, are recommended per the local prescribing information for TMZ.

Best supportive care and treatment should be given as appropriate to each patient (antibiotics, transfusions, oxygen therapy, nutritional support, palliative treatment for pain or cough, etc.).

5. TREATMENT ASSESSMENT

The patient will be evaluated while on treatment in clinic as outlined in section 4.2. MRI scan will be performed every 8 weeks.

The primary assessment of tumor response will be based on clinical status and MRI scans as outlined in the Summary of the Proposed RANO Response Criteria and Criteria for Response Assessment Incorporating MRI and Clinical Factors.

6. ADVERSE EVENTS

The physician will monitor each patient for clinical and laboratory evidence of adverse events on a routine basis while on the expanded access protocol. The physician will assess and record any adverse event in detail in the patient's clinic chart.

6.1 Adverse events definition:

An adverse event is defined as any untoward medical occurrence in a subject or clinical investigation subject administered a pharmaceutical product and which does not necessarily have a causal relationship with this treatment. An adverse event can therefore be any unfavorable and unintended sign (including an abnormal laboratory finding), symptom, or disease temporally associated with the use of a medicinal (investigational) product, whether or not the event is considered causally related to the use of the product.

Such an event can result from use of the drug as stipulated in the protocol or labeling, as well as from accidental or intentional overdose, drug abuse, or drug withdrawal. Any worsening of a pre-existing condition or illness is considered an adverse event. Worsening in severity of a reported adverse event should be reported as a new adverse event. Laboratory abnormalities and changes in vital signs are considered to be adverse events only if they result in discontinuation from the program, necessitate therapeutic medical intervention, meet protocol specific criteria and/or if the doctor considers them to be adverse events.

6.2 Serious Adverse Events (SAE):

A SAE an adverse event that meets any of the following criteria:

**Death of Patient**	An event that results in the death of the patient.
**Life-Threatening**	*An event that, in the opinion of the physician, would have resulted in immediate fatality if medical intervention had not been taken. This does not include an event that would have been fatal if it had occurred in a more severe form.*
Hospitalization or Prolongation of Hospitalization	*An event that results in an admission to the hospital for any length of time or prolongs the patient's hospital stay. This does not include an emergency room visit or admission to an outpatient facility.*
**Congenital Anomaly**	*An anomaly detected at or after birth or any anomaly that results in fetal loss.*
Persistent or Significant Disability/Incapacity	*An event that results in a condition that substantially interferes with the activities of daily living of the patient. Disability is not intended to include experiences of relatively minor medical significance such as headache, nausea, vomiting, diarrhea, influenza, and accidental trauma (e.g., sprained ankle).*
**Important Medical Event Requiring Medical or Surgical Intervention to Prevent Serious Outcome**	*An important medical event that may not be immediately life-threatening or result in death or hospitalization, but based on medical judgment may jeopardize the patient and may require medical or surgical intervention to prevent any of the outcomes listed above (i.e., death of patient, life threatening, hospitalization, prolongation of hospitalization, congenital anomaly, or persistent or significant disability/incapacity). Additionally, any elective or spontaneous abortion or stillbirth*

is considered an important medical event. Examples of such events include allergic bronchospasm requiring intensive treatment in an emergency room or at home, blood dyscrasias or convulsions that do not result in inpatient hospitalization, or the development of drug dependency or drug abuse.

All SAE will be reported to AbbVie within 24 hours and Northwestern University will submit a MedWatch form to the FDA.

6.2.1 Reporting to the FDA

The FDA will be notified no later than 7 calendar days of any SAE that is unexpected fatal or life-threatening.

The FDA will be notified no later than 15 calendar days of any SAE that is serious but not fatal or life-threatening.

All other SAEs will be reported on an annual basis as part of the annual FDA report.

6.3 ABT-414 Toxicity Management:

Based upon results from the preclinical safety pharmacology evaluation of ABT-414, experience with other inhibitors of EGFR, and experience with MMAF antibody drug conjugates, potential toxicities may include fatigue, vomiting, thrombocytopenia, allergic reactions, rash, visual changes, and liver function test abnormalities. Please refer to the current version of the Investigator's Brochure for a complete list of adverse events.

While the physician's discretion should be used for patient management with regards to toxicities, some suggested guidelines are included in the following sections.

6.3.1 Allergic Reactions

The patient will be closely monitored for treatment-related adverse events, especially allergic reactions during all infusions. For the initial ABT-414 infusion, pre infusion vital signs should be taken and direct observation is required for the first 15 minutes of the infusion. Also for the initial ABT-414 infusion, patient must remain at the site for monitoring for 60 minutes post infusion. For subsequent infusions, post-infusion monitoring is not required; however, pre-infusion vital signs should still be taken. Longer observation periods and more frequent vital sign checks may be required in patients who experience infusion reactions.

Institutional standards should be used to treat all allergic reactions.

6.3.2 Severe Allergic Reactions (Grade 3 or Grade 4)

These require the immediate interruption of ABT-414 treatment and permanent discontinuation from the treatment. Appropriate medical therapy including epinephrine, corticosteroids, intravenous antihistamines, bronchodilators, and oxygen should be available for use in the treatment of such reactions. Patients should be carefully observed until the complete resolution of all signs and symptoms.

6.3.3 Moderate Allergic Reactions (Grade 1 or Grade 2)

These will also require the immediate interruption of ABT-414 treatment. Once symptoms have resolved, retreatment is allowed with an infusion over 60 to 70 minutes. All subsequent infusions will also be administered over 60 to 70 minutes.

6.3.4 Dermatologic Toxicities

Patients developing dermatologic toxicities while receiving ABT-414 should be monitored for the development of inflammatory or infectious sequelae, and appropriate treatment of these symptoms initiated. In patients with mild and moderate (Grade 1 or Grade 2) skin toxicity, treatment should continue without dose modification. Treatment with topical and/or oral antibiotics should be considered.

If a patient experiences severe (Grade 3 or Grade 4) acneiform rash, ABT-414 treatment adjustments should be made based on the discretion of the doctor.

Patients who experience another Grade 3 or higher dermatological toxicity will be treated per the following: The patient will require a dose interruption and ABT-414 may be reintroduced at a reduced dose (see section 4.4) if the toxicity returns to ≤ Grade 1 within 4 weeks.

6.3.5 Ophthalmologic Toxicities

All patients should receive prophylactic administration of steroid ophthalmic solution for each administration of ABT-414 as outlined in Section 4.4.

Patients developing ophthalmologic toxicities despite the use of prophylactic steroids must have an examination by an ophthalmologist, including a thorough slit-lamp exam, to evaluate for the presence of epithelial microcysts. The patient should be given steroid ophthalmic solution to use until the toxicity has completely resolved. For patients with mild or moderate symptoms (Grade 1 or 2), the treatments with ABT-414 can continue without dose modification. For patients with severe symptoms (Grade 3 or 4), the administration of ABT-414 must be held until the toxicity has resolved to Grade 1 or better. Re-treatment can be considered at the discretion of the doctor and must be at least one dose level below the dose where the toxicity was observed.

6.4 Pregnancy:

The risks of ABT-414 investigational drug in pregnancy are not known.
If the patient is female and of childbearing potential, or if the patient is male and non-sterile, the patient agrees to practice with partner(s) at least one of the methods of birth control listed in Appendix A beginning with initial ABT-414 administration and continuing until 65 days (for women) or 125 days (for men) after last dose of ABT-414.

In the event of a positive pregnancy test, the patient must immediately discontinue the investigational treatment. The physician must report the positive pregnancy test to the AbbVie representative within 1 working day of the physician becoming aware of the pregnancy.

Pregnancy in a patient's partner must be reported to AbbVie within 1 working day of the physician becoming aware of the pregnancy. Information regarding the occurrence of pregnancy in the patient's partner and the outcome of the pregnancy will be collected.

Pregnancy in patient's partner is not considered an adverse event. However, the medical outcome of an elective or spontaneous abortion, stillbirth or congenital anomaly is considered a serious adverse event and must be reported to AbbVie within 24 hours of the physician becoming aware of the event.

7. DRUG INFORMATION

7.1 Formulation and Packaging:

Identity of Investigational Product

	ABT-414
Dosage Form	Lyophilized powder for reconstitution and intravenous
admixture Formulation	Vial
Strength (mg)	100
Strength (mg)	20

Vials containing ABT-414 lyophilized powder will be packaged in cartons. Each vial and carton

will be labeled per country requirements.

7.2 Preparation and Dispensing:

Refer to the detailed guidelines on Appendix B

7.3 Drug Administration:

ABT-414 will be supplied by AbbVie.

ABT-414 will be supplied vials containing ABT-414 lyophilized powder packaged in cartons. Vials will be supplied in 100 mg or 20 mg strength.

Patient will be administered ABT-414 intravenously once every 2 weeks at a starting dose of 1.25 mg/kg.

Each bottle will be labeled according to requirements and all labels will remain affixed to the bottles.

AbbVie requires that any remaining Investigational Product be destroyed at treatment end. AbbVie will be notified when the treatment period has ended and when the remaining Investigational Product has been destroyed.

7.4 Drug Storage and Drug Accountability:

The ABT-414 lyophilisate for injection must be stored refrigerated at 2°C to 8°C/36°F to 46°F, protected from light, and must not be frozen.

The reconstituted ABT-414 should be refrigerated at 2°C to 8°C/36°F to 46°F, for no more than 20 hours. After storage at 2°C to 8°C/36°F to 46°F, the solution can be allowed to come to room temperature and be administered within 4 hours. From start to reconstitution until the infusion is completed, a total of 24 hours should not be exceeded. If maintained at room temperature, the solution should be used within 4 hours. Reconstitution should be completed per the ABT-414 Study Medication Preparation Guidelines (Appendix B).

Storage temperature logs will be maintained to document proper storage conditions. The refrigerator temperature must be recorded on a daily basis on the temperature logs to record proper function. Temperature excursions must be reported to AbbVie immediately.

The investigational drug is for use only within the context of this expanded access program. ABT-414 supplied for this program must be maintained under adequate security and stored under the conditions specified on the label until dispensed for patient use or returned to AbbVie.

The physician or his/her representative will verify that investigational drug (ABT -414) supplies are received intact and in the correct amounts. This will be documented by signing and dating the Proof of Receipt or similar document. The physician or his/her designated representatives will administer investigational drug only to patients enrolled in this expanded access protocol. A current (running) and accurate inventory of investigational drug will be kept by the physician and will include shipping invoices and the date on which investigational drug is administered to patients. An overall accountability of the investigational drug may be performed and verified by AbbVie during the expanded access program. Upon completion or termination of the program, all original containers containing unused investigational drug (empty containers will be defaced and discarded on site) will be returned to the drug destruction facility according to instructions from AbbVie and according to local regulations. Labels must remain attached to the containers. If pre-arranged between AbbVie and the site, destruction of investigational drug may be performed at the site.

7.5 Chemistry, Manufacturing, and Controls Information:

Enclosed is a Letter of Authorization provided by AbbVie Inc. allowing cross reference to all information in their Investigational New Drug (IND) 115080 for ABT-414 including chemistry, manufacturing facility, pharmacology, and toxicology information.

8. COMPLIANCE WITH GOOD CLINICAL PRACTICE

8.1 Good Clinical Practice:

This treatment will be conducted in compliance with IRB informed consent regulation and the ICH-GCP Guidelines. In addition, all local regulatory requirements will be adhered to, in particular those that afford greater protection to the safety of the trial participants.

This treatment will be conducted according to the current revision of the Declaration of Helsinki (revised Fortaleza, Brazil, 2013) and with local laws and regulations relevant to the use of new therapeutic agents in the country of conduct.

Before initiating the treatment for this patient, the doctor/institution will have written and dated approval/favorable opinion from the IRB for the trial protocol/amendment(s), written informed consent form, and written information to be provided to the patient.

Changes to the protocol will require written IRB approval/favorable opinion prior to implementation, except when the modification is needed to eliminate an immediate hazard(s) to patients.

8.2 Institutional Review:

This expanded access protocol and patient informed consent form will be reviewed and approved by an IRB complying with the requirements of 21 CFR 56 before beginning treatment of patients with investigational ABT-414.

8.3 Informed Consent:

The doctor, or a person designated by the doctor, will explain the benefits and risks of participation in the treatment to this patient, the patient's legally acceptable representative or impartial witness, and obtain written informed consent prior to the patient entering the treatment. The final informed consent form will be agreed to by the sponsor before it is submitted to the IRB and will contain all required elements, in language readily understood by the patient. The original consent form will be signed and dated by the patient or by the patient's legally acceptable representative and also by the person who conducted the informed consent discussion. The doctor will retain the original signed consent form and a copy of the fully executed consent will be provided to the patient.

9. REFERENCES

1. Preusser M, de Ribaupierre S, Wohrer A, et al. Current concepts and management of glioblastoma. Ann Neurol. 2011;70(1):9-21.

2. Stupp R, Mason WP, van den Bent MJ, et al. Radiotherapy plus concomitant and

adjuvant temozolomide for glioblastoma. N Engl J Med. 2005;352(10):987-96.

3. Mason WP, Del Maestro R, Eisenstat D, et al. Canadian recommendations for the treatment of glioblastoma multiforme. Curr Oncol. 2007:14(3):110-7.

4. Yung WK, Albright RE, Olson J, et al. A phase II study of temozolomide vs. procarbazine in patients with glioblastoma multiforme at first relapse. Br J Cancer. 2000;83:588-93.

5. Hegi ME, Diserens A-C, Gorila T, et al. MGMT gene silencing and benefit from temozolomide in glioblastoma. N Engl J Med. 2005;352(10):997-1003.

6. Mellinghoff IK, Wang MY, Vivanco I, et al. Molecular determinants of the response of glioblastomas to EGFR kinase inhibitors. NEJM. 2005, 353(19):2012.

7. Pelloski CE, Ballman KV, Furth AF, et al, Epidermal growth factor receptor variant III status defines clinically distinct subtypes of glioblastoma. JCO. 2007;25(16):2288.

Appendix A. Birth Control Methods

If the patient is female and of childbearing potential or if the patient is a non-sterile male they must practice at least one of the following methods of birth control with partner(s) beginning with initial dose of ABT-414 and continuing until 65 days (for women) or 125 days (for men) after last dose of ABT-414.

- Total abstinence from sexual intercourse as the preferred life style of the patient; periodic abstinence is not acceptable;
- Surgically sterile partner(s); acceptable sterility surgeries are: vasectomy, bilateral tubal ligation, bilateral oophorectomy or hysterectomy;
- Intrauterine device (IUD);
- Double-barrier method (contraceptive sponge, diaphragm or cervical cap with spermicidal jellies or cream AND a condom);
- Hormonal contraceptives (oral, parenteral or transdermal) for at least 3 months prior to drug administration;

If hormonal contraceptives are used, the specific contraceptive must have been used for at least 3 months prior starting ABT-414. If the patient is currently using a hormonal contraceptive, she should also use a barrier method from initial dose of ABT-414 to 65 days after the last dose of ABT-414. Any contraception method must be continued for 30 days after ABT-414.

Female patient needs to be postmenopausal for at least 1 year to be considered not to be of childbearing potential.

Appendix B. ABT-414 Dosing Preparation Guidelines for Intravenous Infusion

All preparation steps for reconstitution and dose preparation must be performed using proper aseptic IV admixture technique for handling hazardous drugs.

- Store ABT-414 vials in the provided cartons, refrigerated at 2° to 8°C (36° to 46°F) until time of use. DO NOT FREEZE.
- Where possible, medication will be stored on the middle shelf of the refrigerator and must not touch the back of the unit.
- ABT-414 does not contain a preservative. Therefore, the storage of the reconstituted material and the storage of material diluted in the infusion bag should be limited as much as possible. Details of shelf life limits are shown below.
- Temperature excursions must be reported to the AbbVie Global Drug Supply immediately. Should a temperature excursion be identified, maintain the impacted medication at the proper storage conditions, in quarantine, until further instructions are received from AbbVie.
- ABT-414 must be kept in locked secure storage, accessible only to those individuals authorized to prepare and dispense the medication.
- The preparation work area should be clean and clear of unrelated material prior to and during ABT-414 medication preparation and dispensing.

Storage of Medication and Shelf Life of Reconstituted and Diluted Solution

**Supplied by AbbVie**		
**Component**	**Storage Conditions**	**Precautions**

ABT-414 Lyophilisate for Injection 100 mg and 20 mg Vial	*2° to 8°C (36° to 46°F) Refrigerate. DO NOT FREEZE.*	*Store vials in provided cartons until use, protect from light.*
**Prepared By Site**		
**Component**	**Storage Conditions**	**Shelf Life**
ABT-414 reconstituted	*2° to 8°C (36° to 46°F)*	*If you do not intend to use*

solution	*Refrigerated*	*immediately, store the reconstituted solution at 2° to 8°C and use within 20 hours. Note that stability of the final solution (see below) is calculated from the time of vial reconstitution.*
ABT-414 reconstituted and further diluted in sterile Sodium Chloride 0.9% (Normal Saline) (NS) solution for intravenous infusion	*2° to 8°C (36° to 46°F) Refrigerated*	*If you do not intend to use the diluted solution in Normal Saline immediately, store the bag at 2° to 8°C, for no more than 20 hours. After storage at 2° to 8°C, the solution can be allowed to come to room temperature and be administered within 4 hours.* **From start of reconstitution until the infusion is completed, a total of 24 hours should not be exceeded.** **Exposure to room temperature, including drug administration, should not exceed 4 hours**

Safety

These guidelines provide minimum recommended safeguards for medication preparation and dispensing.

- Wear appropriate personal protective equipment.
- Greater containment may be achieved if doses are prepared in a vertical laminar air flow hood.
- When handling ABT-414, follow your site's procedures for handling, disposal, and spill clean-up of hazardous drugs and equipment waste.
- For additional ABT-414 hazard information, consult the Material Safety Data Sheet (MSDS) provided to you the AbbVie personnel.

For technical guidance on safe handling practices, follow local regulations. For example,

in the US, refer to the OSHA Technical Manual **TED 01-00-015, Section VI, Chapter 2**.

http://www.osha.gov/dts/osta/otm/otm_vi/otm_vi_2.html

List of Preparation Materials/Equipment

All supplies are to be provided by the site.

All required ancillary equipment is listed below.

Item No.	Description
1	*Protective gloves*
2	*Protective eyewear*
3	*100 mL Sodium Chloride 0.9 % (Normal Saline) sterile solution for infusion bag*
4	*50 mL Sodium Chloride 0.9 % (Normal Saline) sterile solution for infusion bag (may only be used if the resulting IV bag concentration of study drug in a 100 mL bag would be less than 0.3 mg/mL)*
5	*3 mL sterile syringe with 1/10 mL Graduation and luer-lock*
6	*5 mL sterile syringe with 1/5 mL Graduation and luer-lock*
7	*10 mL sterile syringe with 1/5 mL Graduation and luer-lock*
8	*20 mL sterile syringe with 1 mL Graduation and luer-lock*
9	*30 mL sterile syringe with 1 mL Graduation and luer-lock*
10	*50 mL sterile syringe with 1 mL Graduation and luer-lock*
11	*21G or larger bore needle ** with plastic hub*
12	*Sterile tubing for administration*
13	*Catheter*
14	*Stop cock*
15	*Sterile in-line Intravenous Filter (low protein binding; 0.2 µm)*
16	*ABT-414 Lyophilisate for Injection 100 mg vial*
17	*Sterile Water for Injection, without preservatives (SWFI)*
18	*Isopropyl Alcohol swabs*

**Please note that needles with bores smaller than 21 Gauge may pose a risk to drug product potency.

Preparation and Infusion of Dose Solution

Assemble:

- ABT-414 Lyophilisate for Injection
- 5 mL syringe(s)

- 21G needle(s) or larger bore must be used
- SWFI (Sterile Water for Injection), without preservatives
- Alcohol swabs

1. Swab the top of each vial with alcohol swabs.

2. Reconstitute each vial with the appropriate amount of SWFI. Inject the water slowly through the stopper into the vial.

3. Gently swirl each vial in a circular motion to dissolve the lyophilisate. DO NOT SHAKE OR INVERT THE VIAL. Wait for at least 5 minutes. If the solution is still foaming, allow each vial to stand for another 10 minutes to allow settling. The reconstitution times may vary. Record starting time of reconstitution of the vial.

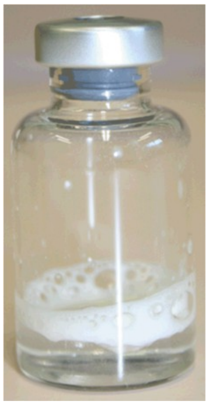
After a few minutes

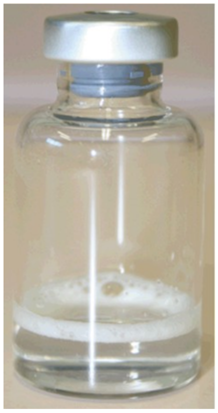
After 10 minutes

4. Visually inspect the solution. Do not use the vial if there is evidence of particles or undissolved material.

5. Add "Use By Date/Time" information to vial label if not used to make IV bag immediately after reconstitution.

Preparation of ABT-414 Infusion Bag Assemble:

- Stage the reconstituted vials from Section 3.2
- 21G needle(s) or larger bore must be used.
- 100 mL Sodium Chloride 0.9% (Normal Saline) sterile solution for infusion bag must be used (If the resulting IV bag concentration of study drug in a 100 mL bag would be less than 0.3 mg/mL a 50 mL Normal

Saline bag must be used.)

- Select syringes of appropriate size according to the required Drug Volume.

1. With the appropriate syringe and needle, withdraw the reconstituted ABT-414 solution.
2. Repeat for each vial. Expel the air bubbles from the syringe.
3. Attach a new capped 21G or larger bore needle to the syringe and check for visible particles.
4. Adjust the drug volume as your calculations.
5. With appropriate syringe and needle, aseptically remove the volume of Normal Saline solution from the Normal Saline bag equivalent to the calculated volume. Discard properly.
6. Inject the ABT-414 solution from the syringe Step (3) into the bag. Gently invert the bag several times to mix the contents. Do not shake.
7. Place the appropriate, label on the IV bag. Record "Use by Date/Time" on label.

Infusion of ABT-414 Solution

- Stage the bag prepared under Section 3.3
- IV line
- In-line or add-on filter (low protein binding 0.2 μm) must be used
- Stop cock, if required

1. Prime the IV line and in line filter with Normal Saline.
2. Attach the prepared ABT-414 infusion bag to the line.
3. Refer to the study protocol for directions on the appropriate infusion time. An infusion time of 3 hours should not be exceeded.
4. Infuse until the ABT-414 infusion bag is completely empty. Record infusion start time.
5. After the infusion is complete, detach the bag, flush the IV line and filter with Normal Saline and discard properly. Record infusion end time.

APPENDIX C- PROTOCOL SUMMARY OF CHANGES

Amendment 1 (FDA Response) – August 3, 2017			
**Section(s) Affected**	**Prior version**	**Amendment 1**	**Rationale**
Cover Page	*n/a*	*Includes IND Number: 136218*	*New information available*
2.1 (Primary Objective)	*The primary object is to provide ABT-414 under the Expanded Access Program for a patient with recurrent GBM who has EGFR amplification or has an EGFRvIII mutation*	*The primary object is to provide ABT-414 under the Expanded Access Program for 5 -10 patients with recurrent GBM who has EGFR amplification or has an EGFRvIII mutation.*	*Clarification requested by FDA*
3 (Patient Eligibility)	*Patients may be enrolled if they meet all of the Inclusion Criteria and do not meet any of the Exclusion Criteria.*	*Up to 10 patients may be enrolled if they meet all of the Inclusion Criteria and do not meet any of the Exclusion Criteria.*	*Clarification requested by FDA*
3.1 (Inclusion criteria)	*n/a*	*Adds the following note: "The patient has adequate hematologic and renal function as follows:"*	*Clarification requested by FDA*
3.1 (Inclusion criteria)	*n/a*	*Adds the following note: "Absolute neutrophil count (ANC) ≥ 1,500/mm³;"*	*Clarification requested by FDA*
3.1 (Inclusion criteria)	*n/a*	*Adds the following note: "Platelets ≥ 100,000/mm³ when administering ABT-414 in combination with temozolomide;"*	*Clarification requested by FDA*
3.1 (Inclusion criteria)	*n/a*	*Adds the following note: "Platelets ≥ 75,000/mm³ when administering ABT-414 monotherapy;"*	*Clarification requested by FDA*

3.1 (Inclusion criteria)	*n/a*	*Adds the following note: "Hemoglobin ≥ 9.0 g/dL (transfusion to achieve Hemoglobin ≥ 9.0 g/dL is acceptable);"*	*Clarification requested by FDA*

3.1 (Inclusion criteria)	*n/a*	*Adds the following note: "Serum creatinine ≤ 1.5 times the upper limit of the normal range"*	*Clarification requested by FDA*
3.1 (Inclusion criteria)	*n/a*	*Adds the following note: "Patient is receiving stable or decreasing doses of steroids for 7 days prior to enrollment"*	*Clarification requested by FDA*
3.2 (EGFR Amplification Testing)	*n/a*	*Tumor samples will be tested for EGFR amplification and expression at Northwestern pathology laboratories. The tumor sample must test positive for EGFR amplification in order for the patient to be eligible for enrollment in the expanded access protocol.*	*Clarification requested by FDA*
3.3 (Exclusion criteria)	*n/a*	*Adds the following note: "Patient has active uncontrolled infection"*	*Clarification requested by FDA*
4.1 (Pre-Treatment Evaluation)	*n/a*	*Adds the following note: "Neurologic Examination"*	*Clarification requested by FDA*
4.1 (Pre-Treatment Evaluation)	*n/a*	*Adds the following note: "EGFR expression"*	*Clarification requested by FDA*

4.1 (Pre-Treatment Evaluation)	*n/a*	*A baseline ophthalmology exam will be performed for all patients prior to initiating treatment. This exam will include the following: external examination, visual acuity, amplitude of accommodation, color vision, cover test, stereopsis, near point of convergence, extraocular motilities, pupils, visual field screening (Humphrey Field Test if available) and interpupillary distance. Other testing may also be performed at the discretion of the examining physician. Additional exams may be performed during the treatment as clinically indicated (e.g., symptoms of dry eyes). In particular, patients developing ophthalmologic toxicities must have an examination by an*	*Clarification requested by FDA*

		ophthalmologist, including a thorough slit-lamp exam, to evaluate for the presence of epithelial microcysts.	
4.2 (Evaluation During Treatment)	*n/a*	*Adds the following note: "Neurologic Examination - Monthly"*	*Clarification requested by FDA*
4.2 (Evaluation During Treatment)	*Ophthalmology exam - As needed*	*Ophthalmology exam - As clinically indicated*	*Clarification requested by FDA*
4.3 (Temozolomide Dosing and Treatment Administration)	*n/a*	*Temozolomide will be administered orally once per day for 5 consecutive days (Days 1 – 5) of a 28-day cycle or as per local prescribing information. The starting dose for the first cycle will be 150 mg/m²/day. The dose of temozolomide can be escalated to 200 mg/m²/day in the second cycle and subsequent cycles.*	*Clarification requested by FDA*
4.6 (Off-Treatment Evaluation)	*n/a*	*Adds the following note: "Neurologic Examination"*	*Clarification requested by FDA*
4.6 (Off-Treatment Evaluation)	*Ophthalmology exam (as needed)*	*Ophthalmology exam (as clinically indicated)*	*Clarification requested by FDA*
**Amendment 2 – November 20, 2017**			
**Section(s) Affected**	**Prior version**	**Amendment 2**	**Rationale**

3.4 (Registration)	*n/a*	*Added a section on registration procedures.*	*Added to clarify the process for enrolling subjects onto this expanded access protocol.*
4.1 (Pre-Treatment Evaluation)	*No timeframes or windows listed for screening procedures.*	*Adds timeframe prior to treatment that evaluations are required; specifies windows if applicable.*	*Added for clarity.*
4.2 (Evaluation During Treatment)	*Missing some timeframes for evaluations; no windows included.*	*Adds or updates timeframes for required evaluations; adds windows if applicable.*<br><br>*Specifies that patients must meet ANC and platelet criteria for treatment prior to each infusion.*	*Added for clarity.*
4.3 (Concomitant Therapy Administration)	*Included option to treatment with concomitant temozolomide only per treating physician's discretion.*	*Adds that treating physician may choose to administer ABT-414 in combination with other standard of care therapies if deemed appropriate and approved by the PI and Abbvie medical monitor.*<br><br>*Adds language for standard of care administration of concomitant bevacizumab.*	*Added per PI decision based on needs of this population.*
4.4 (ABT-414 Dosing and Treatment Administration)	*Stated post-infusion monitoring not required. Did not specify dose modifications allowed.*	*Revised to require post-infusion monitoring after initial ABT-414 infusion (not required for subsequent infusions if no allergic reaction noted). Adds reference to toxicity management section. Adds dose modification table.*	*Clarification and to be consistent with other sections of the protocol.*
4.5 (Off Treatment Criteria)	*n/a*	*Added criteria that were mentioned in other sections of the protocol but not clearly captured in this section.*	*Clarity*
4.6 (Off-Treatment Evaluation)	*No timeframe for off-treatment visit mentioned.*	*Clarifies that evaluations should take place approximately 30 days (+/- 7 days) after last infusion of ABT-414.*	*Clarity*
4.7 (Duration of Treatment)	*n/a*	*This contained duplicate/overlapping information with Section 4.5; information has been removed and consolidated with Section 4.5.*	*Administrative change only.*

4.8 (Other Concomitant Medications)	*Prohibited other anti-cancer treatments other than temozolomide.*	*Adds that treating physician may administer other standard of care agents as appropriate with approval from the PI and Abbvie.*	*Added per PI decision based on needs of this population.*
5 (Treatment Assessment)	*Contained references to another protocol and duplicate information.*	*Removed references to other protocol and any information already described elsewhere.*	*Administrative change only.*
6 (Adverse Events)	*n/a*	*Removed references to other protocols and corrected minor inconsistencies and language regarding management of allergic reactions that was not applicable to this protocol.*	*Administrative change only.*
**Amendment 3 – April 05, 2018**			
Section 2.1 and 3.0	*Maximum number of subjects to be enrolled was 10*	*Maximum number of subjects to be enrolled increased to 20*	*Administrative change*
**Amendment 4 – Jan 08, 2019**			
Section 2.1 and 3.0	*Maximum number of subjects to be enrolled was 20*	*Maximum number of subjects to be enrolled increased to 40*	*Administrative change*

Supplemental Table 1. Demographic and clinical characteristics.

Characteristic	N = 29
**Age at Registration (Years)**	
Median [IQR]	55.0 [51.0, 59.0]
Range	36.0, 68.0
**Sex**	
Female	9 (31.0%)
Male	20 (69.0%)
**Race**	
White	26 (89.7%)
Black	1 (3.4%)
Unknown	2 (6.9%)
**Ethnicity**	
Hispanic or Latino	1 (3.4%)
Non-Hispanic	25 (86.2%)
Unknown	3 (10.3%)
**Region**	
NMH	29 (100.0%)

[1] n (%)

Supplemental Figure 1. Progression free survival (PFS).

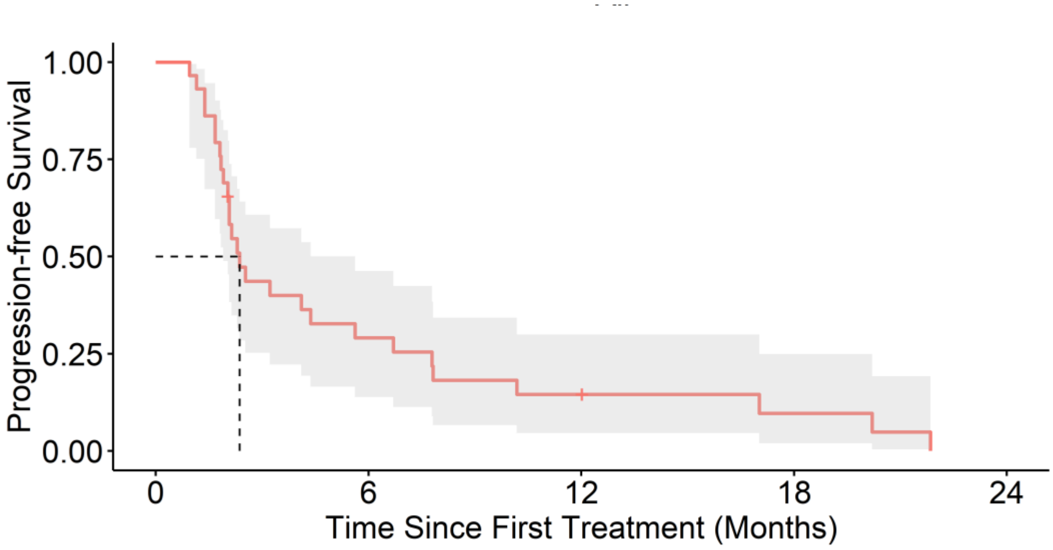

Supplemental Figure 2. Percentage of perivascular EGFR+ cells and progression free survival (PFS).
Scatter plot depicting percent of EGFR+ cells within perivascular regions (40 µm boundary) and corresponding PFS. Blue line represents line of best fit with summary statistics provided.

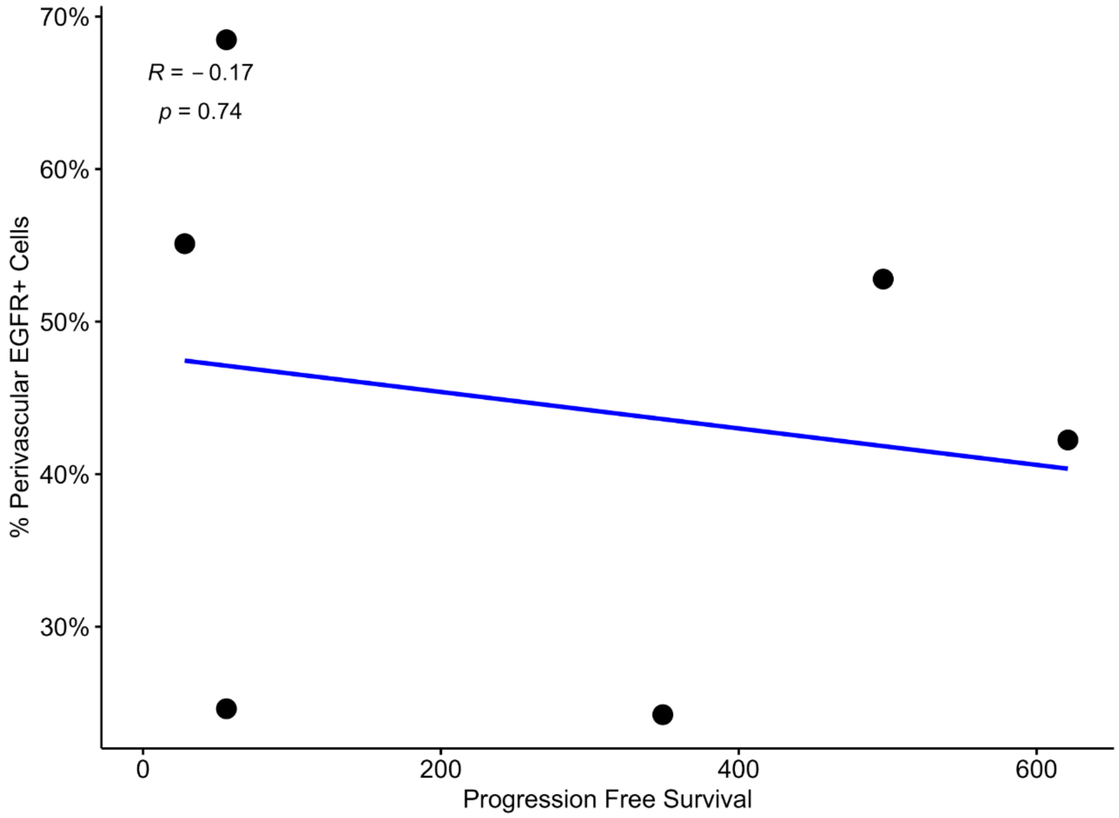

Supplement: Supplemental data [file jciinsight-11-198475-s053.pdf]
